# Enlarged perivascular spaces and white matter hyperintensities in patients with frontotemporal lobar degeneration syndromes

**DOI:** 10.3389/fnagi.2022.923193

**Published:** 2022-07-28

**Authors:** Ming-Liang Wang, Zheng Sun, Wen-Bin Li, Qiao-Qiao Zou, Peng-Yang Li, Xue Wu, Yue-Hua Li, the 4-Repeat Tau Neuroimaging Initiative and the Frontotemporal Lobar Degeneration Neuroimaging Initiative

**Affiliations:** ^1^Department of Radiology, Shanghai Jiao Tong University Affiliated Sixth People’s Hospital, Shanghai, China; ^2^Division of Cardiology, Pauley Heart Center, Virginia Commonwealth University, Richmond, VA, United States; ^3^Institute for Global Health Sciences, University of California, San Francisco, San Francisco, CA, United States

**Keywords:** enlarged perivascular spaces, white matter hyperintensities, PSP-RS, CBS, bvFTD, nfvPPA, svPPA

## Abstract

**Objective:**

The aim of this study was to investigate the distribution characteristics of enlarged perivascular spaces (EPVS) and white matter hyperintensities (WMH) and their associations with disease severity across the frontotemporal lobar degeneration (FTLD) syndromes spectrum.

**Methods:**

This study included 73 controls, 39 progressive supranuclear palsy Richardson’s syndrome (PSP-RS), 31 corticobasal syndrome (CBS), 47 behavioral variant frontotemporal dementia (bvFTD), 36 non-fluent variant primary progressive aphasia (nfvPPA), and 50 semantic variant primary progressive aphasia (svPPA). All subjects had brain magnetic resonance imaging (MRI) and neuropsychological tests, including progressive supranuclear palsy rating scale (PSPRS) and FTLD modified clinical dementia rating sum of boxes (FTLD-CDR). EPVS number and grade were rated on MRI in the centrum semiovale (CSO-EPVS), basal ganglia (BG-EPVS), and brain stem (BS-EPVS). Periventricular (PWMH) and deep (DWMH) were also graded on MRI. The distribution characteristics of EPVS and WMH were compared between control and disease groups. Multivariable linear regression analysis was performed to evaluate the association of EPVS and WMH with disease severity.

**Results:**

Compared with control subjects, PSP-RS and CBS had more BS-EPVS; CBS, bvFTD, and nfvPPA had less CSO-EPVS; all disease groups except CBS had higher PWMH (*p* < 0.05). BS-EPVS was associated with PSPRS in PSP-RS (β = 2.395, 95% CI 0.888–3.901) and CBS (β = 3.115, 95% CI 1.584–4.647). PWMH was associated with FTLD-CDR in bvFTD (β = 1.823, 95% CI 0.752–2.895), nfvPPA (β = 0.971, 95% CI 0.030–1.912), and svPPA (OR: 1.330, 95% CI 0.457–2.204).

**Conclusion:**

BS-EPVS could be a promising indicator of disease severity in PSP-RS and CBS, while PWMH could reflect the severity of bvFTD, nfvPPA, and svPPA.

## Introduction

Frontotemporal lobar degeneration (FTLD) is a group of heterogeneous neuropathological disorders causing a wide spectrum of syndromes, including behavioral variant frontotemporal dementia (bvFTD), non-fluent variant primary progressive aphasia (nfvPPA), semantic variant primary progressive aphasia (svPPA), progressive supranuclear palsy Richardson’s syndrome (PSP-RS), and corticobasal syndrome (CBS) (Bang et al., 2015; Murley et al., 2020). The most common pathological protein aggregates in FTLD include microtubule-associated protein tau, TAR DNA-binding protein 43 (TDP-43), RNA-binding protein fused in sarcoma (FUS), as well as other rare proteinopathies.

With the advances in imaging techniques, several neuroimaging methods showed promise in the diagnosis and assessment of FTLD (Peet et al., 2021). At present, regional brain atrophy is frequently used in disease diagnosis and assessment (Bruun et al., 2019; Staffaroni et al., 2020). However, regional brain atrophy is non-specific and appears late in their respective pathological process. 18F-flortaucipir PET imaging has been used to detect the underlying tau or TDP-43 pathology but lacked sensitivity and specificity (Tsai et al., 2019; Ghirelli et al., 2020). A recent anti-tau drug for PSP-RS randomized-controlled trial has been suggested to be ineffective possibly because of late or wrong target (Höglinger et al., 2021). Therefore, it is important to identify other early imaging biomarkers to facilitate diagnosis, clinical trials assessment, and reveal disease mechanisms to find possible treatment targets.

Enlarged perivascular spaces (EPVS) and white matter hyperintensities (WMH) are two common small vessel disease imaging findings (Wardlaw et al., 2013). Previous studies have shown the effect of WMH on prognosis, course of Alzheimer’s disease (Lee et al., 2016), Parkinson’s disease (Lee et al., 2020), and cognitive impairment (Hu et al., 2021), suggesting the involvement of not only vascular risk factors but also possible dementia-related pathological processes. A recent study also found increased WMH in bvFTD compared with normal control (Huynh et al., 2021). A further study revealed different WMH burden and regional distribution across the FTLD syndromes (Desmarais et al., 2021), but the study sample was small with only 10–20 two subjects in each group.

Compared with WMH, EPVS were less studied and the clinical implication of EPVS remained controversial. Traditionally, EPVS was regarded as an imaging marker of small vessel disease and correlated with aging (Doubal et al., 2010). Perivascular space has been suggested to play an important part in glymphatic system, through which brain waste products are cleared from the brain (Iliff et al., 2012; Rasmussen et al., 2018). Impairment of the glymphatic system may lead to perivascular space enlargement and reduce the clearance of brain waste products, which would further cause retrograde enlargement of perivascular space (Venkat et al., 2017). Several studies have found associations between EPVS and neurodegenerative diseases, including Alzheimer’s disease (Banerjee et al., 2017), Parkinson’s disease (Li et al., 2020; Donahue et al., 2021; Shen et al., 2021), Fabry disease (Lyndon et al., 2021), and Huntington’s disease (Chan et al., 2021). A previous study even found an association between EPVS and brain tau deposition in normal older population (Wang et al., 2021) and early AD continuum (Vilor-Tejedor et al., 2021). To the best of our knowledge, no specific study has investigated the distribution characteristics and clinical value of EPVS across the FTLD syndromes.

In this study, we hypothesize that brain EPVS and WMH burden would increase in subjects with FTLD syndromes compared with control subjects. As the pathological changes of PSP and CBS were mostly found in brain stem (BS) and basal ganglia (BG), while the pathological changes of bvFTD, nfvPPA and svPPA were mostly located in cortex, we anticipated that brainstem EPVS (BS-EPVS) and basal ganglia EPVS (BG-EPVS) load would be most evident in PSP and CBS, while centrum semiovale EPVS (CSO-EPVS) would be most evident in bvFTD, nfvPPA, and svPPA. We also hypothesize the EPVS number and WMH would correlate with disease severity reflected by neuropsychological tests. Our study would enhance the understanding of the disease mechanism and provide potential early imaging biomarkers for disease severity assessment.

## Materials and methods

### Study subjects

The data used in this study were obtained from two databases: The 4R Tau Neuroimaging Initiative (4RTNI) and the Frontotemporal Lobar Degeneration Neuroimaging Initiative (FTLDNI). The 4RTNI is directed by Adam Boxer, M.D., Ph.D., University of California San Francisco (UCSF), and sponsored by the National Institutes of Health (NIH) and the Tau Research Consortium. The FTLDNI is directed by Howard Rosen, MD, Neurology, UCSF, and funded by the National Institute on Aging (Primary) and the National Institute on Neurological Disorders and Stroke. Written informed consent was obtained from all the participants, and the protocol was approved by the local institutional review boards of all participating sites. To be noted, the collected clinical information and the MRI imaging parameters for 4RTNI are the same as those for FTLDNI. Thus, the controls recruited through FTLDNI can be used for comparisons with 4RTNI data. For up-to-date information, please visit http://4rtni-ftldni.ini.usc.edu/.

All subjects aged between 45 and 90 completed brain MRI examination, including 3D-T1WI, 3D-T2WI, and 3D-Flair Sequence, and a series of neuropsychological tests data, including progressive supranuclear palsy rating scale (PSPRS) and FTLD modified clinical dementia rating sum of boxes (FTLD-CDR). The controls were healthy aging people and normal in cognitive status with a CDR of 0. The time interval between MRI and neuropsychological tests was within 1 month.

Subjects were excluded from the projects if they had (1) any significant psychiatric or neurological disease, including major depression, bipolar disorder, schizophrenia, Parkinson’s disease, multi-infarct dementia, Huntington’s disease, stroke, brain tumor, seizure disorder, subdural hematoma, multiple sclerosis, or history of significant head trauma or known structural brain abnormalities; (2) any significant systemic illness or unstable medical condition; (3) long-standing history of history of alcohol or substance abuse; and (4) contraindications to undergoing MRI.

### Clinical assessment

The demographics, including age, sex, and education years, were recorded. We also collected the neuropsychological test results, including Mini-Mental State Examination (MMSE), California Verbal Learning Test (CVLT), FTLD-CDR, PSPRS, and Schwab and England Activities of Daily Living Scale (SEADL). PSPRS was used to assess the disease severity of PSP-RS and CBS (Golbe and Ohman-Strickland, 2007; Dutt et al., 2016). The disease severity of bvFTD, nfvPPA, and svPPA was evaluated by FTLD-CDR (Knopman et al., 2008).

### Magnetic resonance imaging data acquisition and analysis

The MRI data analyzed in this study were all conducted at the UCSF and Massachusetts General Hospital, all of which used a 3 Tesla scanner with Siemens Tim Trio system (Siemens, Iselin, NJ) equipped with a 12-channel receiver head coil. The procedures and parameters for each site were identical. The acquisition parameters were as follows: (1) T1WI: Repetition time ms/echo time ms/inversion time ms, 2,300/3/900; fip angle, 9°; thickness, 1 mm; matrix, 240 × 256; (2) T2WI: repetition time ms/echo time ms, 3,200/403; fip angle, 120°; thickness, 1 mm; matrix, 256 × 256; (3) T2-flair: repetition time ms/echo time ms/inversion time ms, 6,000/389/2,100; fip angle, 120°; thickness, 1 mm; matrix, 256 × 256.

**FIGURE 1:**
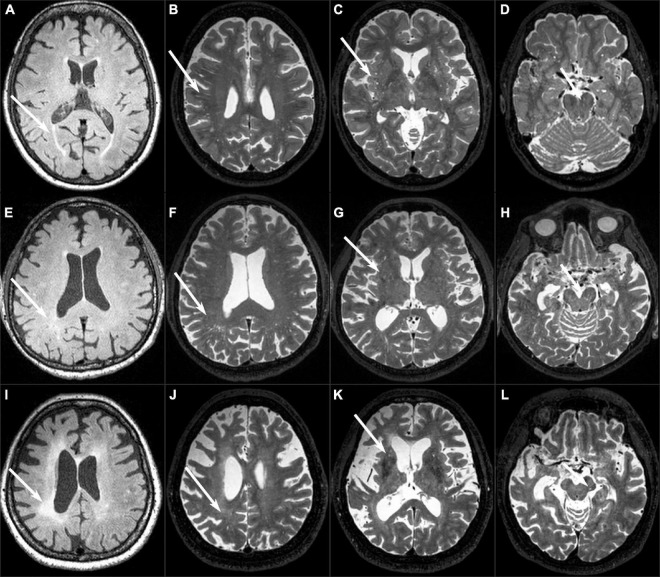
Examples of EPVS and WMH. **(A–D)** A normal 82-year-old female control subject had grade 1 PWMH, grade 3 CSO-EPVS, grade 2 G-EPVS, and two BS-EPVS. **(E–H)** An 85-year-old male patient with PSP-RS had grade 1 PWMH, grade 3 CSO-EPVS, grade 2 BG-EPVS, and 11 BS-EPVS. **(I–L)** A 63-year-old female patient with bvFTD had grade 3 PWMH, grade 1 CSO-EPVS, grade 1 BG-EPVS, and 1 BS-EPVS.

Two trained neuroradiologists (MLW and WBL with 10 and 24 years of experience, respectively) who were blinded to the clinical and neuropsychological test results assessed the MRI data according to the Standards for Reporting Vascular Changes on Neuroimaging (Wardlaw et al., 2013). EPVS were located along the penetrating arteries and characterized as low signals on T1-weighted and FLAIR images and high signal on T2-weighted images. EPVS were rated in the BS, centrum semiovale (CSO), and BG regions. The largest number of EPVS was recorded on one slice of one side of the brain in the CSO region and BG region. For the region of BS, the EPVS were counted within all slices in the whole BS. The connecting EPVS in continuous slices should be counted only once. The CSO-EPVS and BG-EPVS were further assessed with a validated five-point rating scale (0 = no EPVS, 1 = 1–10 EPVS, 2 = 11–20 EPVS, 3 = 21–40 EPVS, and 4 = 40 or more EPVS) (Maclullich et al., 2004; Potter et al., 2015; Figure 1).

WMH lesions were segmented by the lesion prediction algorithm as implemented in the LST toolbox version 3.0.0 for Statistical Parametric Mapping 12.^1^ WMH was also graded in the periventricular region (PWMH) and deep white matter region (DWMH) using the Fazekas rating scale (Fazekas et al., 1987) as they may have different roles in the FTLD. PWMH was defined as contiguous with the ventricle, while DWMH was defined as not contiguous with the ventricle. Severe WMH was defined as having a score > 1 of the Fazekas scale. The total intracranial volume (TIV) was calculated from 3D-T1-weighed image using FMRIB’s Automated Segmentation Tool (Zhang et al., 2001).

The inter-rater reliability (*n* = 100 randomly selected scans) was excellent for the CSO-EPVS number [intraclass correlation coefficient (ICC) = 0.83, 95% CI 0.76–0.88], BG-EPVS number (ICC = 0.85, 95% CI 0.79–0.90), BS-EPVS number (ICC = 0.88, 95% CI 0.83–0.92), PWMH (κ = 0.89, 95% CI 0.85–0.93), and DWMH (κ = 0.88, 95% CI 0.84–0.93). The intra-rater reliability was determined from a random sample of 50 subjects with a 1-month interval between the first and second image assessments assessed by one neuroradiologist. The intra-rater reliability was also excellent for the CSO-EPVS number (ICC = 0.85, 95% CI 0.75–0.91), BG-EPVS number (ICC = 0.88, 95% CI 0.80–0.93), BS-EPVS number (ICC = 0.90, 95% CI 0.83–0.94), PWMH (κ = 0.91, 95% CI 0.86–0.96), and DWMH (κ = 0.89, 95% CI 0.83–0.95). The EPVS rating scores of the senior radiologist were used for the analysis.

### Statistical analyses

Data are expressed as mean (standard deviation) for continuous variables and as frequencies (percentage) for categorical variables. Participants’ demographics and neuropsychological test results between control group and each disease groups were compared using the chi-square test for qualitative variables and the *t*-test or analysis of covariance (ANCOVA) test for quantitative variables, as appropriate. ANCOVA adjusting for age, sex, education years, and TIV was used to examine group differences in WMH and EPVS. *Post hoc* multiple comparisons were done with Bonferroni test. Multivariable linear regression analysis was performed to explore the associations of EPVS and WMH with disease severity reflected by PSPRS in PSP-RS and CBS, FTLD-CDR in bvFTD, nfvPPA, and svPPA. Age, sex, education years, and TIV were used as covariates for all the regression analyses. *p* < 0.05 were considered statistically significant. All statistical analysis was performed using Statistical Package for Social Sciences (IBM Corp., Armonk, NY, United States) for Windows, version 20.0. Graphs were plotted using the GraphPad Prism 8.0 software (GraphPad Software, Inc., United States).

## Results

### Clinical characteristics of the study subjects

A total of 39 subjects with PSP-RS and 31 subjects with CBS in the 4RTNI database, 47 subjects with bvFTD, 36 subjects with nfvPPA, 50 subjects with svPPA, and 73 normal controls in the FTLDNI database were included in this study.

The clinical characteristics of the study subjects are shown in Table 1. As the study subjects came from two databases, there were statistical differences in age (*p* < 0.001), education years (*p* = 0.001), and MMSE (*p* = 0.004) between the control and disease groups. Furthermore, there was also a significant difference in FTLD-CDR among disease groups (*p* < 0.001) and in PSPRS between PSP-RS and CBS (*p* = 0.019).

**TABLE 1:** Participant demographics and clinical characteristics.

	Control (*n* = 73)	PSP-RS (*n* = 39)	CBS (*n* = 31)	bvFTD (*n* = 47)	nfvPPA (*n* = 36)	svPPA (*n* = 37)	*p*-value
Age, years	63.21 (7.16)	69.28 (7.22)^a^	65.81 (6.21)	61.23 (6.74)^bc^	68.06 (7.26)^ad^	62.95 (6.17)^be^	<0.001
Sex, male (%)	29 (39.7)	18 (46.2)	12 (38.7)	30 (63.8)	16 (44.4)	21 (56.8)	0.103
Education, years	17.39 (1.88)	15.38 (4.28)^a^	16.70 (4.20)	15.18 (2.91)^ac^	16.00 (2.57)^a^	16.31 (2.69)	0.001
MMSE	27.03 (3.75)	24.92 (4.15)^a^	23.93 (5.86)^a^	23.98 (4.50)^a^	25.79 (4.03)^a^	25.06 (4.87)^a^	0.004
FTLD-CDR		3.64 (2.52)	3.47 (3.13)	6.36 (2.98)^bc^	2.08 (2.07)^bcd^	3.64 (2.00)^de^	<0.001
CVLT-recall		21.50 (6.04)	22.69 (7.19)	21.14 (7.53)	22.32 (6.91)	17.82 (6.53)^cd^	0.136
PSPRS		35.79 (16.30)	27.23 (12.80)				0.019
SEADL		53.78 (27.01)	56.55 (22.08)				0.777

PSP-RS, progressive supranuclear palsy Richardson’s syndrome; CBS, corticobasal syndrome; bvFTD, behavioral variant frontotemporal dementia; nfvPPA, non-fluent variant primary progressive aphasia; svPPA, semantic variant primary progressive aphasia; MMSE, Mini-Mental State Examination; FTLD-CDR, FTLD modified clinical dementia rating sum of boxes; CVLT, California Verbal Learning Test; PSPRS, progressive supranuclear palsy rating scale; SEADL, Schwab and England Activities of Daily Living. Values are reported as mean (SD) for the continuous variables and as frequency (%) for the categorical variables. ^a^p < 0.05 compared with control group, ^b^p < 0.05 compared with PSP-RS group, ^c^p < 0.05 compared with CBS group, ^d^p < 0.05 compared with bvFTD group, ^e^p < 0.05 compared with nfvPPA group.

### Comparison of enlarged perivascular spaces and white matter hyperintensities among control group and disease groups

**FIGURE 2:**
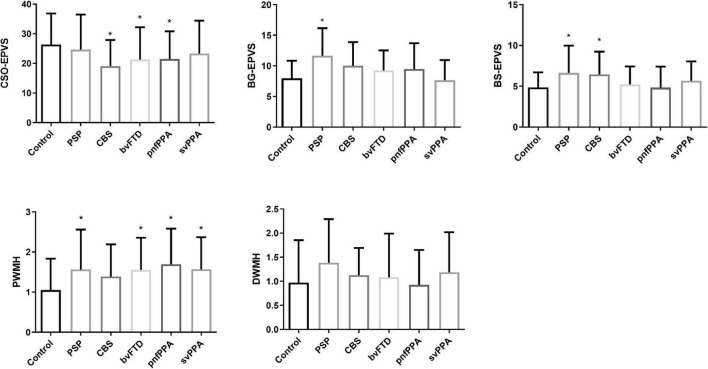
Comparison of EPVS and WMH burden between the control and disease groups. *p < 0.05 compared with control group.

Table 2 and Figure 2 show the distribution characteristics of neuroimaging findings across FTLD syndromes spectrum after controlling for age, sex, education, and TIV. Compared with control subjects, subjects with CBS, bvFTD, and nfvPPA had less CSO-EPVS (*p* = 0.001, 0.011, and 0.007, respectively). Subjects with PSP-RS had more BG-EPVS than control subjects (*p* = 0.002); nfvPPA had fewer BG-EPVS than PSP-RS (*p* = 0.024); svPPA had fewer BG-EPVS than PSP-RS, CBS, and bvFTD (*p* < 0.001, 0.007, and 0.029, respectively). Subjects with PSP-RS and CBS had more BS-EPVS than control subjects (*p* = 0.049 and 0.004, respectively); nfvPPA had fewer BS-EPVS than PSP-RS, CBS, bvFTD, and svPPA (*p* = 0.006, 0.001, 0.034, and 0.018, respectively).

**TABLE 2:** Prevalence of EPVS and WMH across all groups.

	Control (*n* = 73)	PSP-RS (*n* = 39)	CBS (*n* = 31)	bvFTD (*n* = 47)	nfvPPA (*n* = 36)	svPPA (*n* = 37)	*p*-value
CSO-EPVS level							0.063
1	0 (0)	5 (12.8)	5 (16.1)	9 (19.1)	3 (8.3)	4 (10.8)	
2	22 (30.1)	9 (23.1)	11 (35.5)	16 (34.0)	14 (38.9)	12 (32.4)	
3	38 (52.1)	20 (51.3)	15 (48.4)	18 (38.3)	17 (47.2)	17 (45.9)	
4	13 (17.8)	5 (12.8)	0 (0)	4 (8.5)	2 (5.6)	4 (10.8)	
CSO-EPVS number	26.30 (10.51)	24.64 (11.84)	19.10 (8.80)^a^	21.28 (10.91)^a^	21.44 (9.38)^a^	23.35 (11.05)	0.001
BG-EPVS level							<0.001
1	55 (75.3)	13 (33.3)	16 (51.6)	27 (57.4)	22 (61.1)	32 (86.5)	
2	17 (23.3)	25 (64.1)	14 (45.2)	20 (42.6)	13 (36.1)	4 (10.8)	
3	1 (1.4)	1 (2.6)	1 (3.2)	0 (0)	1 (2.8)	1 (2.7)	
BG-EPVS number	7.97 (2.86)	11.64 (4.52)^a^	10.03 (3.84)	9.26 (3.27)	9.47 (4.21)^b^	7.65 (3.28)^bcd^	<0.001
BS-EPVS number	4.85 (1.82)	6.64 (3.34)^a^	6.45 (2.79)^a^	5.21 (2.21)	4.69 (2.66)^bcd^	5.68 (2.37)^e^	<0.001
PWMH level							0.010
0	16 (21.9)	4 (10.3)	2 (6.5)	1 (2.1)	2 (5.6)	1 (2.7)	
1	41 (56.2)	19 (48.7)	19 (61.3)	27 (57.4)	15 (41.7)	20 (54.1)	
2	12 (16.4)	6 (15.4)	6 (19.4)	11 (23.4)	11 (30.6)	10 (27.0)	
3	4 (5.5)	10 (25.6)	4 (12.9)	8 (17.0)	8 (22.2)	6 (16.2)	
Severe PWMH, n (%)	15 (20.5)	16 (41.0)^a^	10 (32.3)	19 (40.4)^a^	19 (52.8)^a^	16 (43.2)^a^	0.017
DWMH level							0.149
0	23 (31.5)	6 (15.4)	3 (9.7)	12 (25.5)	9 (25.0)	6 (16.2)	
1	35 (47.9)	17 (43.6)	21 (67.7)	24 (51.1)	21 (58.3)	22 (59.5)	
2	9 (12.3)	11 (28.2)	7 (22.6)	6 (12.8)	5 (13.9)	5 (13.5)	
3	6 (8.2)	5 (12.8)	0 (0)	5 (10.6)	1 (2.8)	4 (10.8)	
Severe DWMH, n (%)	15 (20.5)	16 (41.0)	7 (22.6)	11 (23.4)	6 (16.7)	9 (24.3)	0.174
WMH volume (cm^3^)	1.46 (2.44)	5.21 (6.23)^a^	3.19 (3.72)	4.98 (6.06)^ac^	3.26 (4.06)^d^	4.21 (6.96)^a^	<0.001
TIV (dm^3^)	1.926 (0.198)	1.895 (0.189)	1.808 (0.267) ^a^	1.854 (0.305)^a^	1.776 (0.412)^ab^	1.901 (0.178)	<0.001

PSP-RS, progressive supranuclear palsy Richardson’s syndrome; CBS, corticobasal syndrome; bvFTD, behavioral variant frontotemporal dementia; nfvPPA, non-fluent variant primary progressive aphasia; svPPA, semantic variant primary progressive aphasia; CSO, centrum semiovale; BG, basal ganglia; BS, brain stem; EPVS, enlarged perivascular spaces; PWMH, periventricular white matter hyperintensities; DWMH, deep white matter hyperintensities; TIV, total intracranial volume. Values are reported as mean (SD) for the continuous variables and as frequency (%) for the categorical variables. Analyses of covariance (ANCOVA) adjusting for age, sex, education years, and TIV was used to examine group differences in WMH and EPVS. ^a^p < 0.05 compared with control group, ^b^p < 0.05 compared with PSP-RS group, ^c^p < 0.05 compared with CBS group, ^d^p < 0.05 compared with bvFTD group, ^e^p < 0.05 compared with nfvPPA group. Post hoc multiple comparisons were done with Bonferroni test.

Compared with control subjects, PSP-RS, bvFTD, and svPPA had more WMH volume (*p* = 0.003, < 0.001, and < 0.001, respectively); bvFTD had more WMH volume than CBS and nfvPPA (*p* = 0.016 and 0.009, respectively). Specially, PSP-RS, bvFTD, nfvPPA, and svPPA had higher PWMH than control subjects (*p* = 0.021, 0.018, 0.001, and 0.012, respectively), while there was no statistical difference in DWMH across all groups. Compared with control subjects, CBS, bvFTD, and nfvPPA had less TIV (*p* = 0.019, 0.004, and 0.001, respectively); nfvPPA had less TIV than bvFTD (*p* = 0.038).

### Associations of enlarged perivascular spaces and white matter hyperintensities with disease severity of each disease group

In multivariable linear regression analysis, BS-EPVS was associated with PSPRS in PSP-RS (β = 2.395, 95% CI 0.888–3.901) and CBS (β = 3.115, 95% CI 1.584–4.647) (Table 3). PWMH was associated with FTLD-CDR in bvFTD (β = 1.823, 95% CI 0.752–2.895), nfvPPA (β = 0.971, 95% CI 0.030–1.912), and svPPA (β = 1.330, 95% CI 0.457–2.204) (Table 4).

**TABLE 3:** Association of EPVS and WMH with PSPRS scores in PSP-RS and CBS.

	β Estimate	Confidence interval (95%)	*p*-value
**PSP-RS**			
CSO-EPVS number	0.153	–0.276 to 0.583	0.472
BG-EPVS number	0.590	–0.470 to 1.651	0.265
BS-EPVS number	2.395	0.888–3.901	0.003*
PWMH level	–1.272	–7.408 to 4.864	0.675
DWMH level	–1.013	–7.188 to 5.161	0.740
**CBS**			
CSO-EPVS number	–0.519	–1.001 to –0.037	0.036*
BG-EPVS number	–0.292	–1.694 to 1.110	0.671
BS-EPVS number	3.115	1.584–4.647	<0.001*
PWMH level	5.332	–1.401 to 12.065	0.115
DWMH level	0.786	–7.985 to 9.556	0.855

PSP-RS, progressive supranuclear palsy Richardson’s syndrome; CBS, corticobasal syndrome; CSO, centrum semiovale; BG, basal ganglia; BS, brain stem; EPVS, enlarged perivascular spaces; PWMH, periventricular white matter hyperintensities; DWMH, deep white matter hyperintensities. Multiple linear regression models were constructed taking PSPRS scores as the outcome, adjusting by age, sex, years of education, and TIV.

*p < 0.05.

**TABLE 4:** Association of EPVS and WMH with FTLD-CDR scores in bvFTD, nfvPPA, and svPPA.

	β Estimate	Confidence interval (95%)	*p*-value
**bvFTD**			
CSO-EPVS number	–0.076	–1.795 to 0.081	0.081
BG-EPVS number	0.102	–0.208 to 0.411	0.573
BS-EPVS number	0.210	–0.232 to 0.652	0.342
PWMH level	1.823	0.752–2.895	0.001*
DWMH level	0.910	–0.180 to 2.000	0.099
**nfvPPA**			
CSO-EPVS number	–0.017	–0.094 to 0.059	0.646
BG-EPVS number	0.029	–0.157 to 0.215	0.755
BS-EPVS number	0.119	–0.167 to 0.404	0.401
PWMH level	0.971	0.030–1.912	0.044*
DWMH level	–0.082	–1.183 to 1.018	0.879
**svPPA**			
CSO-EPVS number	–0.027	–0.102 to 0.048	0.464
BG-EPVS number	0.047	–0.238 to 0.332	0.738
BS-EPVS number	–0.020	–0.380 to 0.339	0.909
PWMH level	1.330	0.457–2.204	0.004*
DWMH level	1.225	0.226–2.224	0.018*

FTLD-CDR, FTLD modified clinical dementia rating sum of boxes; bvFTD, behavioral variant frontotemporal dementia; nfvPPA, non-fluent variant primary progressive aphasia; svPPA, semantic variant primary progressive aphasia; CSO, centrum semiovale; BG, basal ganglia; BS, brain stem; EPVS, enlarged perivascular spaces; PWMH, periventricular white matter hyperintensities; DWMH, deep white matter hyperintensities. Multiple linear regression models were constructed taking FTLD-CDR scores as the outcome, adjusting by age, sex, years of education, and TIV. *p < 0.05.

## Discussion

In this study, we assessed the distribution characteristics of EPVS and WMH across FTLD syndromes and the associations with disease severity. Our study found that EPVS and PWMH discriminated FTLD syndromes from controls, mainly showing that subjects with PSP-RS and CBS had more BS-EPVS, subjects with CBS, bvFTD, and nfvPPA had fewer CSO-EPVS, and all disease groups except CBS had higher PWMH compared with control subjects. BS-EPVS was associated with disease severity in PSP-RS and CBS. PWMH was associated with disease severity in bvFTD, nfvPPA, and svPPA.

Interestingly, we found that subjects with PSP-RS and CBS had more BS-EPVS than control subjects, indicating a possible role in the disease pathophysiology. Most of the PSP-RS and CBS were 4R-tauopathies, characterized by the deposition of tau isoforms with four repeats of the microtubule-binding domain (Stamelou et al., 2021). PSP was characterized by brain pathology in pallidonigroluysian system, followed by the BG, and the frontal and parietal lobes (Williams et al., 2007), while the CBS pathology first appeared in frontoparietal and motor cortical areas and the striatum followed by other subcortical nuclei and the brainstem (Kouri et al., 2011). The excessive misfold tau protein and neuronal debris might stack in the perivascular spaces causing the appearance of EPVS, and malfunction of PVS would further slow the clearance of brain waste, suggesting a feedforward mechanism.

Unexpectedly, the subjects with CBS, bvFTD, and nfvPPA had fewer CSO-EPVS than control subjects. This was somehow contrary to the circumstance of BS-EPVS. CBS, bvFTD, and nfvPPA were all characterized by predominant frontal and/or temporal neurodegeneration and brain shrinkage (Rosen et al., 2002). We speculate that the decreased number of EPVS may represent a consequence of total obstruction of the PVSs, which will cause loss of CSF signal and decreased detectability of EPVS. Furthermore, previous studies have suggested the association between CSO-EPV but not BG-EPVS or BS-EPVS and intracranial volume (Dubost et al., 2019). Severe atrophy of the brain parenchyma may cause the collapse of PVS structures in CBS, bvFTD, and nfvPPA. Thus, the combination of impairment of PVS system and brain atrophy in CBS, bvFTD, and nfvPPA may lead to less CSO-EPVS distribution. A previous study also revealed lower anterosuperior medial temporal lobe PVS in mild cognitive impairment subjects (Sepehrband et al., 2021). Furthermore, the heterogeneous pathology underlying the FTLD syndromes spectrum may cause the different distribution characteristics of EPVS.

In our study, we found that more subjects with FTLD syndromes had severe PWMH than control subjects. This was similar to a recent study indicating more WMH burden in FTLD spectrum (Desmarais et al., 2021). Traditionally, the underlying pathology of PWMH was attributed to hypoperfusion and ischemia commonly found in the elderly population (Kim et al., 2008). In the case of FTLD spectrum, the deposition of insoluble protein along the penetrating arteries may not only aggregate hypoperfusion but also cause impaired interstitial fluid drainage of the brain glymphatic system (Benveniste and Nedergaard, 2021), which could cause axonal degeneration, leading to the higher WMH burden. To be noted, the difference between CBS and control subjects did not reach a statistical difference. This may be caused by small sample size (*n* = 31).

Consistent with our hypothesis, BS-EPVS was associated with disease severity in PSP and CBS. PWMH was associated with disease severity in bvFTD, nfvPPA, and svPPA. Previous studies have revealed the effect of PWMH on cognitive impairment and degenerative processes in the elderly population (van den Heuvel et al., 2006) and dementia diseases (Hu et al., 2021). PWMH mostly affect the long association fibers, which would further cause disconnection of functional related cortical and subcortical structures. The increased BS-EPVS number might reflect the insufficient clearance of FTLD-related pathological protein deposits. Future longitudinal work is needed to figure out the mechanism of BS-EPVS and its involvement in pathological changes and clinical feature progression. Our study suggested that PWMH and BS-EPVS would be useful in reflecting the severity of FTLD syndromes in clinical practice.

Our study should be interpreted in light of the following limitations. First, we have no information about the pathological type of the study FTLD syndromes, and thus the pathology may be heterogeneous. Future correlational study with the pathological changes may lead to a more comprehensive understanding of EPVS and WMH in FTLD syndromes. Second, due to the cross-sectional nature of our study, we cannot determine the exact order of causal effects between EPVS, PWMH, and disease progression. Third, disease duration was not obtained in FTLDNI project. Further investigations are therefore required to assess the influence of disease duration on EPVS and WMH. Fourth, the information on cardiovascular risk factors like hypertension, diabetes, and hyperlipidemia was not available and hence not included as covariables in all the analyses. However, 4RTNI and FTLDNI projects had strict inclusion and exclusion criteria excluding any significant neurological and cardiovascular diseases, which would reduce these potential influences. Finally, we used validated visual rating scales to assess EPVS, which was rater-dependent and has ceiling or floor effects. However, the assessment method was simple and the inter-rater and intra-rater reliability for the rating of EPVS was excellent. The visual rating method could be applied easily in practical clinical practice.

## Conclusion

Our study demonstrated that EPVS and PWMH discriminated subjects with FTLD syndromes from controls. BS-EPVS could be a promising indicator of disease severity in PSP-RS and CBS, while PWMH could reflect the severity of bvFTD, nfvPPA, and svPPA.

## Data availability statement

The datasets presented in this study can be found in online repositories. The names of the repository/repositories and accession number(s) can be found below: http://4rtni-ftldni.ini.usc.edu/.

## Ethics statement

The studies involving human participants were reviewed and approved by the Local Institutional Review Boards of all participating sites. The patients/participants provided their written informed consent to participate in this study.

## Author contributions

M-LW and Y-HL: study concept and design. M-LW and ZS: analysis and interpretation of data and drafting of the manuscript. M-LW, Q-QZ, P-YL, and XW: statistical analysis and critical revision of the manuscript. Y-HL and W-BL: administrative, technical and material support, and study supervision. All authors gave final approval of the version published.

## Footnotes

1
www.statistical-modeling.de/lst.html
